# Establishing a committee for antemortem reviews of suspect Creutzfeldt-Jakob disease cases in Ireland

**DOI:** 10.1007/s11845-022-03070-2

**Published:** 2022-07-15

**Authors:** Conor Fearon, Rachel Howley, Seamus Looby, Amber Byrne, Josephine Heffernan, Ciara Heeney, Alan Beausang, Jane Cryan, Michael Farrell, Sean O’Dowd, Francesca Brett

**Affiliations:** 1 Department of Neuropathology, Beaumont Hospital, Dublin 9, Ireland; 2 Department of Neuroradiology, Beaumont Hospital, Dublin 9, Ireland; 3 Department of Neurology, Tallaght University Hospital, Dublin 24, Ireland; 4 Academic Unit of Neurology, Trinity College, Dublin, Ireland

**Keywords:** Creutzfeldt-Jakob disease, Prion disease

## Abstract

**Background:**

Creutzfeldt-Jakob disease (CJD) is a rapidly progressive, neurodegenerative disease. In Ireland, clinical diagnostics and laboratory testing remain the responsibility of the managing clinician and the Neuropathology Department at the Beaumont Hospital, respectively. Centralized review of individual cases is not undertaken.

**Aims:**

To determine how diagnostic processes for CJD could be improved in Ireland and to outline the structure and referral process for a new CJD review panel at the Beaumont Hospital.

**Methods:**

We surveyed Irish neurologists’ experiences on the management of CJD in Ireland. We measured turnaround times (TAT) for CSF samples referred for diagnostic CJD testing. Finally, we retrospectively reviewed imaging of autopsy-proven CJD cases to compare with initial reports.

**Results:**

Ninety-three percent of neurologists supported a national central review of suspect CJD cases. A second clinical opinion was considered to be of likely benefit by 79%. Additionally, 93% reported that a centralized review of neuroradiology would be useful. All respondents felt that expediting turnaround of CSF analysis would be of benefit. The average TAT for CSF testing was 35.4 days. In retrospective review of imaging, all patients demonstrated MRI findings consistent with CJD. However, in only one of these cases were the initial pre-autopsy radiological findings reported as being consistent with CJD.

**Conclusions:**

These findings support the need for improvements to the Irish National CJD Surveillance Unit to maximize antemortem diagnostic accuracy. On foot of this, a clinical CJD Multidisciplinary Team (CJD MDT) has been established to provide a second opinion on (i) the patient’s clinical history, (ii) neuroradiology and (iii) and neurophysiology reports (where available).

## Introduction

Creutzfeldt-Jakob disease (CJD) is a rapidly progressive and invariably fatal neurodegenerative disease caused by the misfolding of the endogenous prion protein into an abnormal form which culminates in spongiform degeneration of cerebral grey matter. The incidence is approximately 2.2 cases per million population per year with a peak age of onset in the 7th decade [1]. In most cases (approximately 85%), the cause is unknown (sporadic CJD, sCJD). A rarer acquired form (variant CJD, vCJD), which has received significant media coverage, has been associated with dietary exposure to the cattle prion disease, bovine spongiform encephalopathy, although person-to-person transmission of vCJD infection has also been reported [2]. Iatrogenic causes are now rare but previously resulted from treatment with cadaveric pituitary-derived human growth hormone and gonadotrophins as well as cadaver-derived dura mater neurosurgical grafts. Genetic forms of CJD (and other related prion diseases) may account for up to 10–15% of cases [3]. However, a family history may be absent as penetrance is incomplete.

The phenotypic spectrum of sCJD is highly heterogeneous. Although classically a rapidly progressive dementia with ataxia, myoclonus, pyramidal and extrapyramidal signs, many less typical forms of the disease exist and include isolated cognitive impairment (15%), pure ataxic variants (10%), higher order visual disturbances (5%), psychiatric presentations (5%), corticobasal syndrome (2%), stroke-like presentations (2%) and pain/sleep disturbances (2%) [4]. Hence, clinical diagnosis, particularly in the early stages of the illness, may be challenging with definitive diagnosis that can only established at autopsy. When brain biopsy is performed for suspect sCJD, the final neuropathological diagnosis differs from this clinical suspicion in most cases [5]. Although sCJD is the prototypical “rapidly progressive dementia”, many other neurodegenerative disorders may also show rapid clinical progression. In addition, many treatable conditions may mimic sCJD including autoimmune or paraneoplastic limbic encephalitis, lymphoma, primary CNS vasculitis, non-convulsive status epilepticus and toxic/metabolic encephalopathy.

In suspect sCJD, paraclinical data can aid the diagnostic process. When the classical MRI picture of cortical ribboning with striatal (± thalamic) T2/FLAIR and diffusion-weighted imaging changes is seen in the appropriate clinical context, diagnostic confidence should be high. However, in routine clinical practice, these often subtle and symmetric MRI findings are easily overlooked, especially in the early stages of disease [6]. EEG is less sensitive and specific than MRI in detecting sCJD, but periodic sharp wave complexes appear in half to two-thirds of patients with sCJD, usually in the later stages of the disease when clinical suspicion is higher [7]. For this reason, EEG is an essential adjunct to MRI in detecting the presence of alternative diagnoses including non-convulsive status epilepticus, toxic/metabolic encephalopathy as well as viral or autoimmune encephalitis.

For many years, cerebrospinal fluid (CSF) analysis has been used to support a diagnosis of sCJD by detection of proteins released into the CSF in the setting of rapid neurodegeneration (protein 14–3-3, neurone-specific enolase and S100b). Although the sensitivity of these tests is relatively high (> 80%), the specificity for sCJD is much lower with false-positive results occurring in any process where rapid neurodegeneration occurs. The development of the real-time quaking-induced conversion (RT-QuIC) assay for CJD has dramatically changed the landscape of diagnostics in prion diseases [8]. With a sensitivity of 89% and a specificity which asymptotically approaches 100% [9], RT-QuIC has opened the door to a more definitive premortem diagnosis of CJD which has relevance for patients (by obviating a need for further testing) and families (who can benefit from greater certainty in diagnosis at the time of often traumatic and rapid progression to death in their relatives) as well as for clinicians.

In the UK, a National CJD Research & Surveillance Unit (NCJDRSU) was established in 1990. Its aims included the identification of all cases of CJD in the UK and investigation of each case further by clinical examination, clinical investigation, neuropathologic examination, genetic analysis, molecular biological studies and collection of basic epidemiological data [10]. Suspect cases were reviewed during life, and the NCJDRSU neurologist corresponded with the local clinician outlining their clinical formulation and diagnostic classification. A European Creutzfeldt-Jakob Disease Surveillance Network (EuroCJD) and American National Prion Disease Pathology Surveillance Center have also been in place to conduct epidemiological surveillance of CJD since the 1990s [11, 12].

Although a CJD infection control committee exists in Ireland under the oversight of the Health Protection Surveillance Centre, its function is primarily related to the public health and epidemiological aspects of CJD and other transmissible spongiform encephalopathies. The clinical diagnostic aspects and laboratory testing for individual cases remain the responsibility of the managing clinician and the Neuropathology Department at Beaumont Hospital, respectively. Centralized review of individual cases is not undertaken, and the RT-QuIC assay is not currently available in Ireland. CSF samples are therefore currently sent to the UK NCJDRSU for this analysis.

In order to determine how diagnostic processes for CJD could be improved in Ireland, we conducted a review of these processes by:

1. Surveying Irish neurologists’ experiences and opinions on management of CJD in Ireland
2. Measuring turnaround times for CSF samples referred for diagnostic testing for CJD
3. Retrospectively reviewing imaging of autopsy-proven cases of CJD in Ireland to compare findings with initial reports

Finally, we outline the structure and referral process for a new CJD review panel at the Beaumont Hospital.

## Methods

### Survey

An online survey of 9 questions was distributed to all neurologists in Ireland. All responses were collected anonymously in an online database and analysed.

Questions included the following:

1. The nature of the neurology service at their hospital.
2. The number of cases of CJD the clinician has seen over the prior 5 years.
3. Whether a national central review committee for CJD would be beneficial.
4. Whether a second clinical opinion from a neurologist would be beneficial in selected cases.
5. Whether central review of imaging for cases of suspected CJD would be of benefit.
6. Whether expeditious turnaround of CSF for prion diseases would be of benefit.
7. If in their experience, families of patients with suspect CJD are well supported through and after their relative’s diagnosis.
8. Whether a central point of contact for families of patients with suspected CJD during work-up and after diagnosis would be beneficial.
9. Whether provision of a clinical nurse specialist would be an important aspect of such a service.

### Review of CSF turnaround times

Records of all CSF samples referred for diagnostic testing for CJD over a 6-year period (1 January 2015 to 31 December 2020) were audited to ascertain positivity of protein 14–3-3 and RTQuIC and turnaround time (TAT) for reporting of samples sent for analysis to the UK NCJDRSU. TAT was calculated based on the number of calendar days (i.e. including weekends) from date of sample receipt at the Irish National CJD Surveillance Unit (INCJDSU) Beaumont Hospital, until date of report authorization.

### Imaging review

Brain MRIs, where available, of all cases of autopsy-confirmed sporadic CJD from 1 January 2018 to 1 January 2019 were anonymised and reviewed retrospectively by an experienced neuroradiologist who was not blinded as to the final neuropathologic diagnosis. Changes suggestive of CJD were documented and compared to the original reports.

## Results

### Survey

**Fig. 1:**
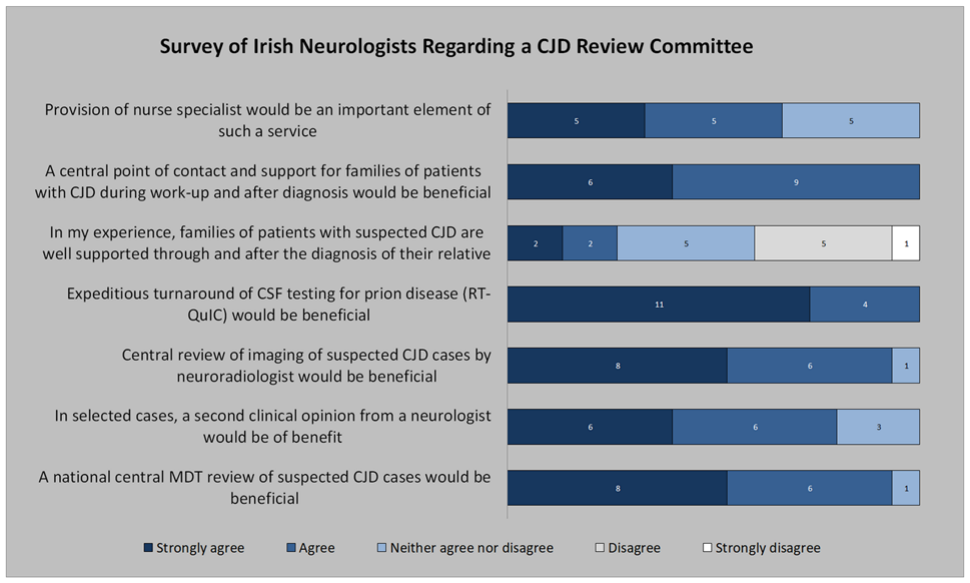
Responses of Irish neurologists to survey on CJD review committee

An online survey was sent to 57 Irish neurologists. Fifteen (26.3%) neurologists responded to the survey. Ninety-three percent worked in hospitals with a full-time neurology service, while 7% worked on a part-time liaison/consultation basis. The mean (± standard deviation) number of CJD cases managed by the respondents over the past 5 years was 2.2 ± 1.38. Their responses are shown in Fig. 1. Ninety-three percent of respondents felt that a national central review of suspected CJD cases would be beneficial. Seventy-nine percent reported that in selected cases, a second clinical opinion from a neurologist would be beneficial, while 93% agreed that centralized review of neuroradiology would be useful. All respondents felt that expediting turnaround of CSF samples for prion testing would be of benefit and that a central point of contact and support for families of patients diagnosed with CJD would be advantageous.

### Review of CSF analysis

**Fig. 2:**
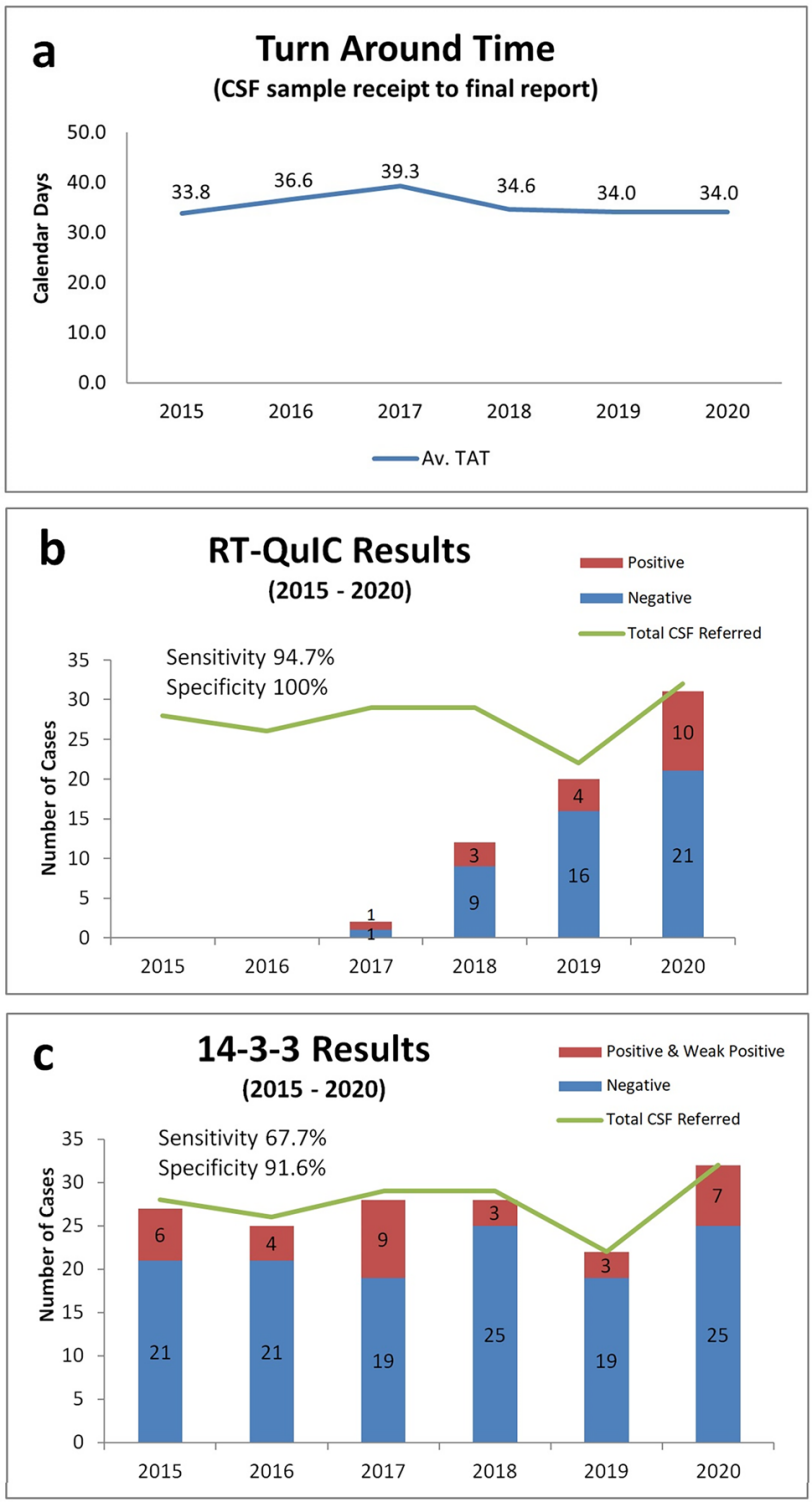
**a** Turnaround time (TAT) in calendar days from CSF sample receipt to report authorization over a 6-year period from 2015 to 2020. The UK NCJDSRU provides this service with a TAT of 14 days from receipt of sample; reporting delays were a consequence of sample batching in Ireland to reduce shipping costs. RT-QuIC results (**b**) and 14–3-3 results (**c**) reported during 2015–2020 represented as a portion of the total CSF samples received, stratified according to positive results (red) and negative results (blue) per annum

A total of 166 CSF samples were received by INCJDSU between 1 January 2015 and 31 December 2020. The mean number of cases referred per annum was 28 (range 22–32). The average TAT was 35.4 calendar days (range 13–147); and as can be seen from Fig. 2a, it remained consistent across the 6-year period, despite the constraints placed on the system by COVID-19 in 2020.

A total of 65/166 CSF cases had RT-QuIC results, 27.7% of which were reported positive. 97.6% (162/166) of the CSF referrals had 14–3-3 analysis performed with 19.75% demonstrating positive or weak positive results. As can be seen in Fig. 2b, RT-QuIC reporting was gradually introduced from 2017, while 14–3-3 analysis was consistently reported throughout the 6-year time period (Fig. 2c).

In order to assess sensitivity and specificity, a review of definite and probable CJD cases identified by the INCJDSU during the same 6-year period was performed. Only 1 false negative was identified among the 65 RT-QuIC samples analysed demonstrating a sensitivity of 94.7% and specificity of 100%. On the other hand, there were 11 false positives and 10 false negatives identified among the 162 CSF samples processed for 14–3-3 analysis resulting in a sensitivity of 67.7% and specificity of 91.6%. Of interest, 38% of the definite/probable cases identified during the 6-year interval were not referred to the INCJDSU for CSF analysis (14–3-3 or RT-QuIC) during life highlighting the need to improve premortem surveillance services.

### Imaging review

**Fig. 3:**
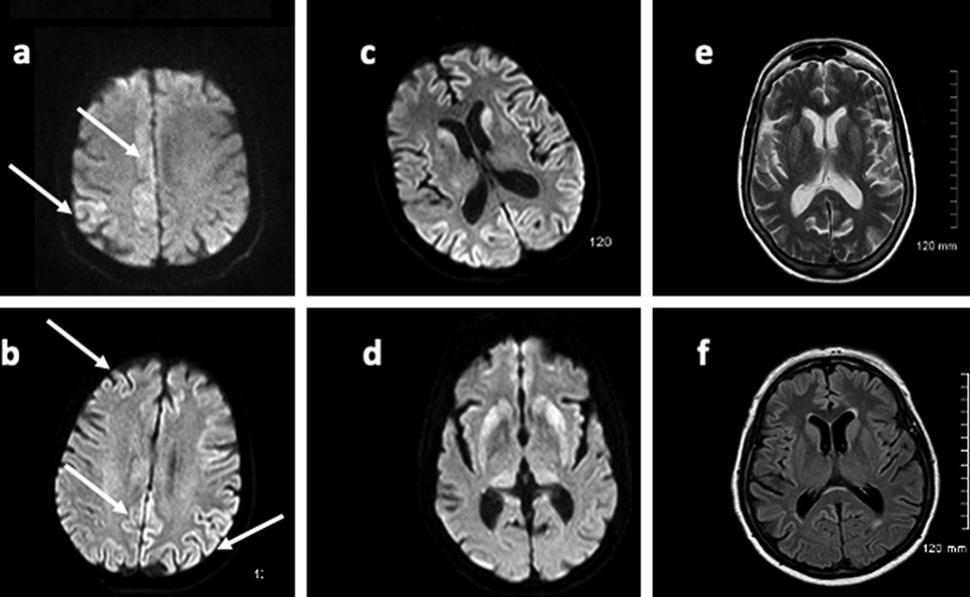
Sample images of premortem MRI brain sequences demonstrate findings suggestive of sCJD. **a**, **b** Diffusion-weighted sequences demonstrating cortical restricted diffusion (“cortical ribboning”) of bilateral parietal, cingulate and right frontal cortices; **c**, **d** diffusion-weighted sequences demonstrating restricted diffusion in bilateral caudate (**c**, **d**) and putamen (**d**), **c** also demonstrates cortical ribboning, **d** also demonstrates bilateral posterior thalamic diffusion restriction—an atypical finding in sCJD; **e** T2 and **f** FLAIR sequences demonstrating bilateral caudate and putamenal hyperintensity

Of the 9 patients with sCJD who underwent post-mortem examination, 8 had premortem MRI imaging performed. All eight studies had at least axial T2, axial FLAIR and diffusion-weighted imaging sequences performed. Findings suggestive of sCJD are shown in Table 1. In retrospective review, all 8 patients demonstrated MRI findings consistent with CJD. However, in only one of these cases were the radiological findings reported as being consistent with CJD. Sample images taken from these cases are shown in Fig. 3. Clinical details are omitted for confidentiality.

**Table 1:** Premortem MRI findings in 8 sequential cases of pathologically confirmed sCJD. Presence and site of T2/FLAIR and DWI abnormalities are shown

**Case no.**	**T2/FLAIR abnormality present**	**Site of T2/FLAIR abnormality**	**DWI abnormality present**	**Site of DWI abnormality**	**MRI reported as suggestive of sCJD at time of reporting**
1	No	-	Yes	Right cingulate gyrus and parietal cortex bilaterally	No
2	Yes	Bilateral caudate nuclei	Yes	Bilateral caudate nuclei	No
3	Yes	Bilateral caudate and lentiform nuclei	Yes	Bilateral caudate and lentiform nuclei	No
4	No	-	Yes	Right cingulate gyrus and bilateral caudate nuclei	No
5	Yes	Right parietal and frontal cortex and bilateral caudate nuclei	Yes	Right parietal and frontal cortex and bilateral caudate nuclei	Yes
6	Yes	Bilateral caudate nuclei and parieto-occipital cortex	Yes	Bilateral caudate nuclei and parieto-occipital cortex	No
7	No	-	Yes	Bilateral thalami, bilateral occipital cortex, left parietal cortex	No
8	No	-	Yes	Bilateral caudate nuclei	No

## Discussion

This review examined specific attitudes to and current processes involved in diagnostics of sporadic CJD in Ireland. Firstly, we found that the majority of neurologists surveyed felt that a centralized CJD clinical review panel would be beneficial, to help reduce turnaround of CSF diagnostic testing, to centralize imaging review and to create a central point of contact for families of patients diagnosed with CJD. Secondly, the turnaround time for CSF testing ranged from 13 to 147 calendar days with a mean of 35.4 days. The majority of this delay was due to the batching of samples to reduce expensive dry ice shipping costs when sending samples to the UK NCJDSRU. Finally, retrospective review of cases of sCJD who had premortem neuroimaging showed that all cases demonstrated changes consistent with CJD which were not reported at the time the scan was performed in all but one case. The MRIs were reviewed by an experienced neuroradiologist, who was already aware that the patient had a post mortem diagnosis of CJD. The authors acknowledge the advantage this gave the reviewing neuroradiologist in reviewing these MRIs. Although not all centres in Ireland have access to experienced neuroradiologists or dedicated MDT meetings, the crucial aspect highlighted by this review is that all patients with rapidly progressive dementia or rapidly progressive ataxia should have a carefully reviewed DWI sequence included in their imaging. The MRI assessment performed here carries an obvious bias. Unblinded retrospective review of imaging where the post-mortem diagnosis of sCJD is known obviously improves detection of the often subtle findings of early sCJD. In three of the cases reviewed, only DWI changes were present; T2/FLAIR sequences were normal. In three of the cases reviewed, only DWI changes were present. T2/FLAIR sequences were normal. The pooled diagnostic yield of diffusion-weighted imaging in sporadic Creutzfeldt-Jakob disease has been reported as high as 91% [13]. The diagnostic performance of diffusion-weighted imaging for predicting sporadic Creutzfeldt-Jakob disease among patients with rapidly progressive dementia had a pooled sensitivity of 91%, and specificity of 97% with simultaneous involvement in the neocortex and striatum, the most common finding on DWI.

**Fig. 4:**
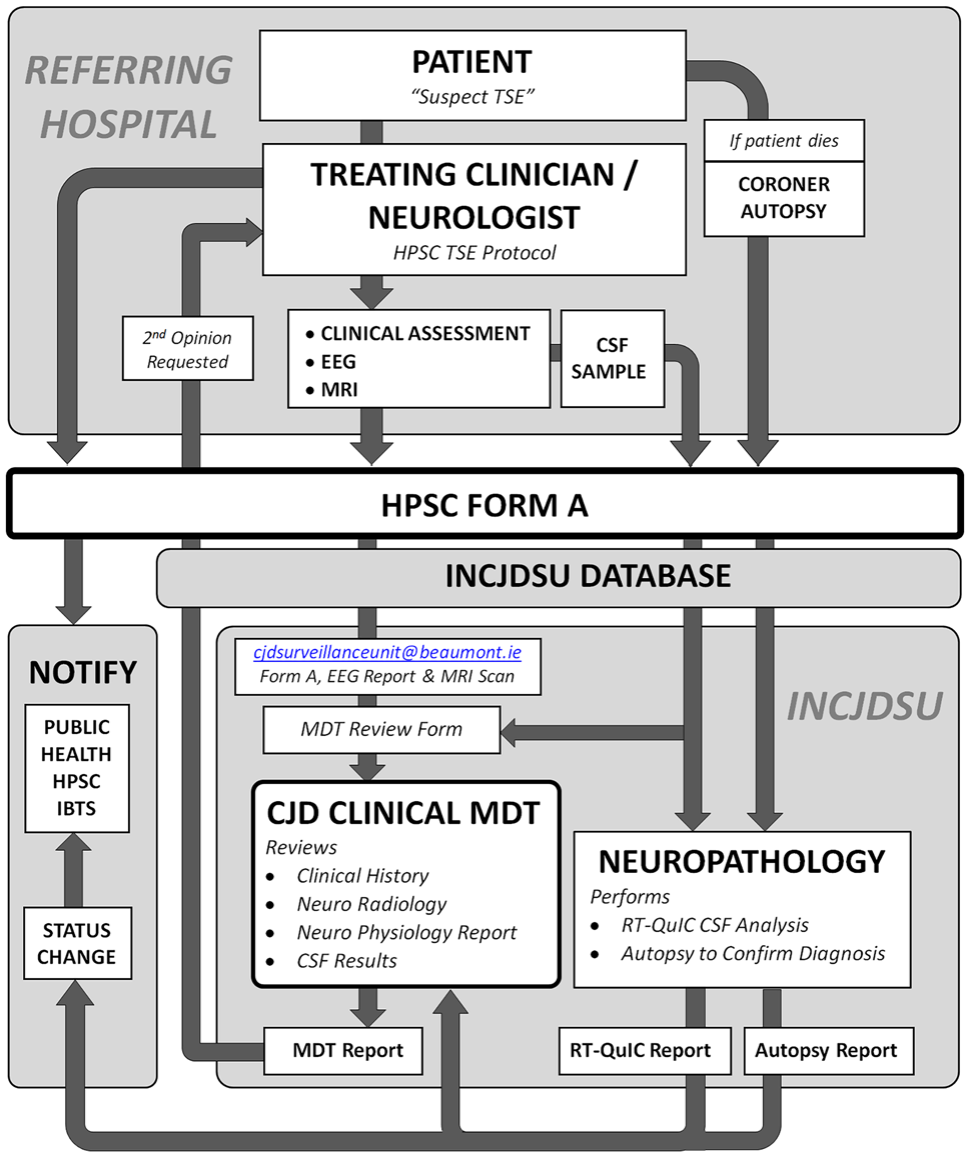
CJD referral process outlining the pathway of reporting and case review from the referring hospital, the Irish National CJD Surveillance Unit (INCJDSU) and the Health Protection Surveillance Centre (HPSC) and Irish Blood Transfusion Service (IBTS)

Nevertheless, together these findings strongly support the need for improvements to the Irish National CJD surveillance unit in order to maximize antemortem diagnostic accuracy of this rare, yet notifiable, disease. On foot of the survey results a clinical CJD Multidisciplinary Team (CJD MDT) that has been established to provide a second opinion on (i) the patient’s clinical history, (ii) neuroradiological scans and (iii) neurophysiology reports (where available). The referral process is outlined in Fig. 4. Clinicians wishing to avail of the CJD MDT service simply complete Form A (Appendix 11) of “Protocol for Reporting and Management of cases of Creutzfeldt Jakob Disease (CJD) and other Transmissible Spongiform Encephalopathies (TSEs) or of a person at increased risk of a TSE” (9). Form A is sent along with the clinical history, EEG report and a copy of MRI scans (if not on the NIMIS system) to the Irish NCJDSU, at the Beaumont Hospital, by email (cjdsurveillanceunit@beaumont.ie) or by post (Irish NCJDSU, Department Neuropathology, Beaumont Hospital, Dublin 9) specifying the requirement for a second opinion. Once the review has been performed, a final MDT report comprising of neurological and neuroradiological review is returned to the managing physician along with any further test results (i.e. CSF) that have been performed.

Furthermore, in order to create an Irish central point of contact for (i) clinicians searching for up-to-date CJD-specific information and (ii) families of patients diagnosed with CJD, a National CJD Surveillance Unit website is now available at https://www.cjd.ie

Given the potential public health issues associated with managing a “Query” CJD patient, the need for an antemortem test with a high degree of sensitivity and specificity to support CJD surveillance is clear. In contrast to 14–3-3 analysis, RT-QuIC is a robust assay which is easy to interpret and is the first laboratory-based test requested when prion disease is considered within the differential diagnosis. Funded by Department of Public Health & the Health Service Executive (HSE), RT-QuIC analysis has now been established in the Irish NCJDSU, Neuropathology Department, Beaumont Hospital. The advantages of which will be numerous. Firstly, it will guarantee all Irish “query” CJD cases will be centralized to one location and further reduce the ambiguity for referring clinicians ensuring questions concerning all “query” Irish CJD cases are redirected to the Irish National CJD Surveillance Unit instead of to the UK. Furthermore, and most importantly, it will vastly reduce reporting turnaround times (from 6 weeks to less than 14 days) as samples will no longer need to be sent in batches to the UK for analysis. Access to results with short turnaround times will allow clinicians to screen CSF samples promptly to address the possibility of CJD in particularly challenging neurological cases.

As a result of the vCJD outbreak, CJD became a notifiable disease across many countries, the result of which has seen improvements to diagnostic methods and surveillance practices for all TSEs. Incorporating these improvements into our own Irish National CJD Surveillance Service is overwhelmingly supported by neurologists across the country.
